# Diabetes severity and the role of leisure time physical exercise on cardiovascular mortality: the Nord-Trøndelag Health study (HUNT), Norway

**DOI:** 10.1186/1475-2840-12-83

**Published:** 2013-06-06

**Authors:** Børge Moe, Liv Berit Augestad, Tom IL Nilsen

**Affiliations:** 1 Department of Human Movement Science, Norwegian University of Science and Technology, Trondheim, Norway

**Keywords:** Diabetes severity, Leisure time physical exercise, Cardiovascular mortality, Epidemiology

## Abstract

**Background:**

Physical activity has been associated with lower cardiovascular mortality in people with diabetes, but how diabetes severity influence this association has not been extensively studied.

**Methods:**

We prospectively examined the joint association of diabetes severity, measured as medical treatment status and disease duration, and physical exercise with cardiovascular mortality. A total of 56,170 people were followed up for 24 years through the Norwegian Cause of Death Registry. Cox proportional adjusted hazard ratios (HRs) with 95% confidence intervals (CI) were estimated.

**Results:**

Overall, 7,723 people died from cardiovascular disease during the follow-up. Compared to the reference group of inactive people without diabetes, people with diabetes who reported no medical treatment had a hazard ratio (HR) of 1.65 (95% CI: 1.34, 2.03) if they were inactive and a HR of 0.99 (95% CI: 0.68, 1.45) if they reported ≥2.0 hours physical exercise per week. Among people who received oral hypoglycemic drugs or insulin, the corresponding comparison gave HRs of 2.46 (95% CI: 2.08-2.92) and 1.58 (95% CI: 1.21, 2.05), respectively.

**Conclusions:**

The data suggest a more favourable effect of exercise in people with diabetes who used medication than in those who did not, suggesting that physical exercise should be encouraged as a therapeutic measure additional to medical treatment.

## Background

Among people with diabetes, the highest risk of death from cardiovascular disease is found in those treated with oral hypoglycemic drugs or insulin [1,2] and in those with longer duration of diabetes [3-6]. The combination of insulin treatment and longer duration of diabetes has been associated with a particular high risk of death from cardiovascular disease [7]. These findings imply that diabetes treatment and duration are important markers for diabetes severity.

Regular physical activity may improve glycaemic control, insulin sensitivity, and cardiovascular risk factors (e.g., blood pressure, lipid profile, and body composition) in people with diabetes [8-11]. It has been reported that the most physically active people with diabetes have approximately half the risk of death from cardiovascular disease, compared to physically inactive people with diabetes, [12,13]. In a recent study, people with diabetes who reported a moderate to high level of leisure time physical exercise had the same risk of death from cardiovascular disease as inactive people without diabetes [14]. However, if the beneficial effect of leisure time physical exercise on cardiovascular mortality among people with diabetes is modified by diabetes severity is not known.

The aim of this prospective follow-up study was therefore to assess whether diabetes severity, measured as medical treatment status and disease duration, influence the role of leisure time physical exercise on cardiovascular mortality.

## Methods

### Subjects

The HUNT Study is a large population-based health survey in Nord-Trøndelag County in Norway. Between 1984 and 1986, all inhabitants aged 20 years or older (85,100) were invited to participate in the first wave of the study (HUNT 1) and a total of 77,216 (90.7%) accepted the invitation, filled in a questionnaire and attended a clinical examination. The participants were given a second questionnaire at the clinical examination to complete at home and return in a pre-stamped envelope. All participants in the HUNT Study gave a written informed consent upon participation and the study was approved by the Regional Committee for Ethics in Medical Research.

For the purpose of the present study, a total of 21,046 participants were excluded at baseline; 4,413 who reported prevalent cardiovascular disease (i.e. angina, myocardial infarction, and/or stroke), 1,003 without sufficient information on diabetes (diagnosis, treatment or duration), 15,227 without information on leisure time physical exercise and 403 without information on potentially confounding factors (i.e. systolic blood pressure, body mass index). After these exclusions, 56,170 participants (27,321 men (mean age= 47.4 years old) and 28,852 women (mean age= 48.5 years old)) were available for follow up on cause of death.

### Follow-up

Individual person time at risk of death was calculated from the date of participation in the HUNT 1 study (1984–86) until the date of death or until the end of follow-up 31^st^ December 2008, whichever occurred first. The mandatory reporting of death to the Cause of Death Registry in Norway constitutes the basis for the coding of underlying cause of death. Deaths were classified according to the International Classification of Disease (ICD-9 and ICD-10). Cardiovascular disease was defined by ICD-9: 390–459 and ICD-10: I00-I99, and ischemic heart disease by ICD-9: 410–414 and ICD-10: 120–125.

### Study variables

A detailed description of selection procedures, questionnaires, and measurements can be found at http://www.ntnu.edu/hunt and in a report by Holmen and colleagues [15]. Briefly, information was collected on a range of lifestyle and health related factors, including medical history, leisure time physical exercise, smoking status, alcohol consumption and education. Height was measured to the nearest centimetre and weight to the nearest half kilogram, and body mass index (BMI) was calculated as weight (kg) divided by the square value of height (m). Blood pressure was measured two times using a mercury manometer and the mean of the two measures was used.

### Diabetes

Participants who answered ‘Yes’ to the question ‘Do you have or have you had diabetes?’ in the first questionnaire, were defined as having diabetes (N=1,105).

The second questionnaire included additional questions to those with known diabetes. People who responded ‘No’ to the questions ‘Do you take tablets for your diabetes?’ and ‘Do you take insulin injections?’ were categorized as people without medication, while people who responded ‘Yes’ to one of the questions were categorized as people with medication. We did not have sufficient power in the statistical analyses to assess the two questions separately. The question ‘When were you first diagnosed with diabetes?’, provided information on diabetes duration.

### Leisure time physical exercise

Information on leisure-time physical exercise was obtained from the second questionnaire. The participants were asked ‘How often do you exercise (on the average)?, By exercise we mean going for walks, skiing, swimming and working out/sports’ with five mutually exclusive response options: 0, <1, 1, 2–3, ≥4 times per week. Those who reported exercising once a week or more were also asked ‘for how long do you exercise each time (average)?’, with four mutually exclusive response options: <15, 15–30, 31–60, >60 min. Based on the information of both frequency and duration of exercise we calculated ‘hours of leisure time physical exercise per week’. The frequency response option 2–3 times per week was counted as 2.5 times and ≥4 per week counted as five times, whereas the duration response options <15, 15–30, 31–60, >60 min were counted as 10, 25, 45 and 75 min, respectively. For the purpose of the statistical analysis, we categorized this variable into three groups. This resulted in 22,885 physically inactive participants, 24,140 participants performing 0.1-1.9 hours of leisure time physical exercise per week and 8,040 participants performing ≥2.0 hours of leisure time physical exercise per week.

### Statistical analyses

A Cox proportional hazard model was used to estimate adjusted hazard ratios (HRs) of death from cardiovascular disease and ischemic heart disease associated with diabetes treatment (with or without oral hypoglycemic drugs or insulin) and duration, and in a separate analysis, to assess the combined association of leisure time physical exercise and diabetes treatment with risk of death. Precision of the estimated hazard ratios was assessed by a 95% confidence interval (CI). All estimated associations were adjusted for possible confounding by attained age (as the time scale) and birth cohort (five years strata). In multivariable models we adjusted for smoking status (never, former, current, unknown), alcohol consumption (0, 1–4, ≥ 5 times last 14 days, total abstainer, unknown), education (<10 years, 10–12 years, >13 years, unknown), body mass index (kg/m^2^) and systolic blood pressure (mmHg).

Analyses of the independent association of diabetes treatment were stratified by sex, whereas analyses of the combined association were adjusted for sex in a pooled sample to increase statistical power. The latter was justified by likelihood ratio tests of interaction with sex in the independent analyses of diabetes treatment (*P* = 0.11) and in the analyses combining diabetes treatment and leisure time physical exercise (*P* = 0.37).

Additional to the analyses combining leisure time physical exercise and diabetes treatment, we conducted analyses of physical activity stratified by diabetes treatment and tested for possible statistical interaction between the two variables in a likelihood ratio test. Moreover, a likelihood ratio test was used to assess whether the results were modified by age at baseline (< 70 years and ≥ 70 years).

We also conducted three sensitivity analyses; first, people with probable type 1 diabetes (i.e. who were diagnosed with diabetes before 40 years of age and reported insulin injections); second, we excluded the first five years of follow up to evaluate possible bias that could arise if people with the most severe diabetes were unable to be physically active; third, we excluded people who reported a moderate or high degree of movement disability.

Departure from the proportional hazards assumption was evaluated by Schoenfeld residuals and graphical procedures (log-log plots). All statistical tests were two-sided, and all analyses were conducted using Stata for Windows, version 11.2 (StataCorp LP, Texas 77845, USA).

## Results

Baseline characteristics of the study population according to diabetes severity are presented in Table 1. During a median follow-up of 23.8 years (1,145,786 person-years), a total of 17,383 people died. Of these, 7,723 people died from cardiovascular disease and 3,518 from ischaemic heart disease. Compared to the 16,633 people who were excluded due to missing data on central variables, the 56,170 included study participants were similar in age (mean age, 49.7 versus 49.1 years), but were less likely to die from cardiovascular disease (age and sex adjusted HR 0.85, 95% CI: 0.81, 0.88). There was no evidence of departure from the proportional hazards assumption for any of the exposure variables under study.

**Table 1:** Baseline characteristics of the study population

	**No diabetes**	**Diabetes**	
**Characteristics**		Without medication*	With medication*
Men (n=27,318)			
No. of participants	26,815	202	301
Mean age at study entry (SD), years	47.0 (16.7)	65.9 (12.3)	62.2 (17.0)
Mean duration of diabetes (SD), years		6.1 (6.7)	11.9 (10.7)
Mean body mass index (SD), kg/m^2^	25.2 (3.2)	26.3 (3.7)	26.2 (3.8)
Mean SBP (SD), mmHg	138.7 (19.7)	155.5 (25.1)	151.1 (25.4)
Hours of leisure time physical exercise per week, %			
Inactive	42.5	47.0	38.2
0.1-1.9 hours	40.5	33.2	35.9
≥ 2.0 hours	17.0	19.8	25.9
Current smoker, %	35.3	31.7	21.0
Alcohol consumption ≥ 5 times last 14 days, %	8.6	7.9	5.3
Women (n=28,852)			
No. of participants	28,250	239	363
Mean age at study entry (SD), years	48.1 (17.0)	67.7 (12.5)	66.7 (13.3)
Mean duration of diabetes (SD), years		5.1 (6.0)	10.5 (8.5)
Mean body mass index (SD), kg/m^2^	24.9 (4.4)	28.5 (5.1)	28.5 (5.4)
Mean SBP (SD), mmHg	135.1 (24.9)	163.0 (28.0)	160.2 (26.4)
Hours of leisure time physical exercise per week, %			
None	40.7	43.5	49.0
0.1-1.9 hours	47.0	43.9	34.2
≥ 2.0 hours	12.3	12.6	16.8
Current smoker, %	31.5	13.8	9.1
Alcohol consumption ≥ 5 times last 14 days, %	3.2	1.0	2.2

Abbreviations: *SBP* Systolic blood pressure.

*Medication refers to oral hypoglycemic drugs or insulin.

Table 2 presents the independent association between diabetes treatment, measured as medical treatment status and duration, with risk of death from cardiovascular disease and ischemic heart disease. Compared to people without diabetes, the adjusted HR for cardiovascular death was 1.59 (95% CI: 1.29, 1.96) among men with diabetes who received no medical treatment, and 2.16 (95% CI: 1.80, 2.60) for men with diabetes who used medication. These associations were slightly stronger in women, although no statistical significant interaction with sex was observed (*P*=0.11). The adjusted HR for cardiovascular death was 1.64 (95% CI: 1.34, 2.00) in diabetic women without medication and 2.85 (95% CI: 2.43, 3.34) for those who used medication. Overall, the corresponding analyses of death from ischaemic heart disease gave slightly stronger associations in both men and women.

**Table 2:** Hazard ratios (HRs) for death from cardiovascular disease associated with diabetes treatment and disease duration

		**Death from cardiovascular disease**			**Death from ischaemic heart disease**		
**Mortality**	**No. of person-years**	**No. of deaths**	HR*	**HR† (95% CI)**	**No. of deaths**	HR*	**HR† (95% CI)**
Men							
No diabetes	545,005	3,802	1.00	1.00 (reference)	1,980	1.00	1.00 (reference)
Diabetes without medication‡	2,553	91	1.67	1.59 (1.29-1.96)	49	1.85	1.73 (1.30-2.31)
Diabetes with medication‡	3,739	120	2.09	2.16 (1.80-2.60)	70	2.47	2.53 (1.99-3.22)
No diabetes	545,005	3,802	1.00	1.00 (reference)	1,980	1.00	1.00 (reference)
Diabetes without medication‡							
<5 years	1,354	49	1.59	1.54 (1.16-2.05)	29	2.03	1.91 (1.32-2.77)
≥5 years	1,199	42	1.78	1.66 (1.22-2.26)	20	1.65	1.53 (0.98-2.38)
Diabetes with medication‡							
<10 years	1,967	71	2.01	2.01 (1.59-2.55)	42	2.40	2.39 (1.76-3.26)
≥10 years	1,772	49	2.22	2.42 (1.82-3.22)	28	2.57	2.77 (1.90-4.04)
Women							
No diabetes	597,934	3,442	1.00	1.00 (reference)	1,297	1.00	1.00 (reference)
Diabetes without medication‡	3,327	100	1.88	1.64 (1.34-2.00)	41	2.00	1.65 (1.21-2.26)
Diabetes with medication‡	4,228	168	3.12	2.85 (2.43-3.34)	81	3.78	3.44 (2.73-4.33)
No diabetes	597,934	3,442	1.00	1.00 (reference)	1,297	1.00	1.00 (reference)
Diabetes without medication‡							
<5 years	2,087	60	1.75	1.61 (1.25-2.09)	23	1.75	1.55 (1.03-2.35)
≥5 years	1,240	40	2.12	1.67 (1.22-2.29)	18	2.45	1.80 (1.12-2.87)
Diabetes with medication‡							
<10 years	2,371	104	3.00	2.74 (2.25-3.35)	45	3.26	2.98 (2.20-4.04)
≥10 years	1,857	64	3.36	3.04 (2.37-3.91)	36	4.71	4.25 (3.03-5.95)

Abbreviations: *CI* confidence interval, *HR* hazard ratio.

* Adjusted for age (as the time scale) and birth cohort (5 years strata).

† Adjusted for age (as the time scale) and birth cohort (5 years strata), smoking (never, former, current, unknown), education (<10 years, 10–12 years, >13 years, unknown), alcohol (0, 1–4, ≥ 5 times last 14 days, total abstainer, unknown), systolic blood pressure, body mass index and hours of leisure time physical exercise per week (inactive, 0.1-1.9 hours, ≥ 2.0 hours).

‡Medication refers to oral hypoglycemic drugs or insulin.

In a sensitivity analysis we excluded 113 participants (39 men and 74 women) with possible type 1 diabetes, but the results remained largely unchanged. The HRs for people with diabetes who used medication was 2.23 (95% CI: 1.83, 2.71) in men and and 2.82 (95% CI: 2.36, 3.37) in women (data not shown). Moreover, tests of interaction suggest a possible modifying effect of age (*P*_interaction_ = 0.01 in men and 0.03 in women), with a slightly weaker association in the oldest age groups (≥70 years). However, stratified analyses showed that all associations between diabetes treatment status and mortality remained statistically significant in both age groups (data not shown).

The association between diabetes duration and mortality are also presented in Table 2, displaying the HRs after subdividing each of the medical treatment categories into two categories of disease duration. Overall, disease duration was not strongly related to mortality from cardiovascular disease, although the HRs were generally slightly stronger in the subgroup of longer duration (i.e. ≥5 years for persons without medical treatment, and ≥10 years for those with medical treatment). The slightly stronger associations with longer duration were somewhat more evident in the analyses of ischemic heart disease.

There was statistical evidence of interaction between diabetes treatment and hours of leisure time physical exercise per week (*P* <0.001), suggesting that the favorable effect of physical activity were larger in people with more severe diabetes. Table 3 shows the combined association of diabetes treatment and hours of leisure time physical exercise per week with death from cardiovascular disease and ischemic heart disease, using inactive people without diabetes as the reference group for all comparisons. In people with diabetes who did not use medication, the adjusted HR for cardiovascular death was 1.65 (95% CI: 1.34, 2.03) for inactive people and 0.99 (95% CI: 0.68, 1.45) for those who reported ≥ 2.0 hours of leisure time physical exercise per week. Among people with more severe diabetes (i.e. those who used medication), the adjusted HR for cardiovascular death was 2.46 (95% CI: 2.08, 2.92) in people who were inactive and 1.58 (95% CI: 1.21, 2.05) for those who reported ≥ 2.0 hours of leisure time physical exercise per week. People with diabetes who exercised 0.1-1.9 hours per week had approximately similar HRs as those who were inactive. A similar pattern was observed for death from ischaemic heart disease, although the difference in HRs between inactive people and the most active were somewhat larger.

**Table 3:** Hazard ratios (HRs) for death from cardiovascular disease and ischaemic heart disease associated with diabetes treatment and leisure time physical exercise

		**Death from cardiovascular disease**			**Death from ischaemic heart disease**		
**Hours of leisure time physical exercise per week**	**No. of person-years**	**No. of deaths**	HR*	**HR† (95% CI)**	**No. of deaths**	HR*	**HR† (95% CI)**
Without diabetes							
Inactive	468,436	3,108	1.00	1.00 (reference)	1,391	1.00	1.00 (reference)
0.1-1.9 hours	514,603	2,759	0.78	0.83 (0.79-0.87)	1,256	0.79	0.86 (0.80-0.93)
≥ 2.0 hours	159,901	1,377	0.77	0.84 (0.79-0.89)	630	0.80	0.88 (0.80-0.97)
Diabetes without medication‡							
Inactive	2,359	95	1.74	1.65 (1.34-2.03)	48	2.04	1.90 (1.42-2.54)
0.1-1.9 hours	2,514	69	1.45	1.44 (1.13-1.83)	30	1.50	1.48 (1.03-2.13)
≥ 2.0 hours	1,008	27	1.06	0.99 (0.68-1.45)	12	1.08	1.02 (0.58-1.80)
Diabetes with medication‡							
Inactive	3,151	140	2.49	2.46 (2.08-2.92)	70	2.91	2.92 (2.29-3.72)
0.1-1.9 hours	3,233	91	2.48	2.64 (2.14-3.25)	57	3.41	3.67 (2.81-4.79)
≥ 2.0 hours	1,583	57	1.49	1.58 (1.21-2.05)	24	1.39	1.49 (0.99-2.23)

Abbreviations: *CI* confidence interval, *HR* hazard ratio.

*Adjusted for age (as the time scale) and birth cohort (5 years strata), sex (male, female).

†Adjusted for age (as the time scale) and birth cohort (5 years strata), sex (male, female), smoking (never, former, current, unknown), education (<10 years, 10–12 years, >13 years, unknown), alcohol (0, 1–4, ≥ 5 times last 14 days, total abstainer, unknown), systolic blood pressure, body mass index.

‡Medication refers to oral hypoglycemic drugs or insulin.

These combined effects were further explored in analyses of leisure time physical exercise stratified by diabetes status and treatment. Compared to being inactive, ≥2.0 hours of physical activity per week was associated with an adjusted HR of 0.84 (0.89-0.89) in people without diabetes, a HR of 0.79 (95% CI: 0.56, 1.11) in people with diabetes who received no medical treatment, and a HR of 0.49 (95% CI: 0.56, 1.11) in people with diabetes who were medically treated (data not shown).

In sensitivity analyses, we excluded the first five years of follow up, and then also people who reported a moderate or high degree of movement disability. Both analyses gave largely similar results as those presented above (data not shown).

## Discussion

In this large population based cohort study, less severe diabetes that was not medically treated was associated with a 60 per cent increased risk of death from cardiovascular disease, whereas more severe diabetes treated with oral agents or insulin was associated with a two-fold increased risk in men and a nearly three-fold higher risk in women. Within each category of treatment status, disease duration was not strongly associated with mortality. Leisure time physical exercise reduced the risk of death from cardiovascular disease in both categories of diabetes severity, but there was evidence of a stronger association in people with more severe diabetes. Corresponding analyses of death from ischaemic heart disease gave slightly stronger associations, although the precision of the estimated associations were lower.

The results of the present study are in agreement with previous prospective studies, showing that people with diabetes treated with oral hypoglycemic drugs or insulin have a higher risk of death from cardiovascular disease than those without medication [1,2]. Several studies have reported a positive association between diabetes duration and cardiovascular mortality [3-7], but we did not find any strong effect of diabetes duration on cardiovascular mortality when diabetes treatment was taken into account. Although there was no statistically significant interaction between sex and diabetes treatment in the present study, the association between diabetes treatment and cardiovascular mortality was somewhat stronger in women than in men. Previous studies have also reported a stronger association of diabetes with cardiovascular mortality in women than in men [16,17].

Findings from observational studies suggest that people with diabetes who are physically active have approximately half the relative risk of death from cardiovascular disease compared to inactive people with diabetes [12,13], although different methods for measuring physical activity make comparisons between studies difficult. The results from the present study suggest a differential effect of physical exercise on cardiovascular mortality by diabetes severity, showing that the favorable effect of exercise was relatively stronger in those who reported medical treatment than in those who did not. The beneficial effect of leisure time physical exercise on cardiovascular mortality in people with diabetes may be explained by the sum of improvements in cardiovascular disease risk factors. These risk factors may be more prevalent in medically treated diabetes. Studies have reported increased cardiovascular mortality associated with hyperglycaemia, hypertension, dyslipidaemia and overweight in people with diabetes [13,18-20], whereas regular physical exercise has been shown to improve the same risk factors [8-11,21]. Moreover, a recent review suggests that regular physical exercise could reduce the use of anti-diabetic drugs in people with type 2 diabetes [22].

The strengths of the present study include the population-based sample, the prospective design, the large number of participants, the long follow-up period and the ascertainment of causes of death through the Cause of Death Registry at Statistics Norway.

Limitations of the study include the assessment of diabetes and leisure time physical exercise with a questionnaire at baseline. The self-reported diagnosis of diabetes in HUNT 1 was validated in a separate study [23], showing that 96.4% of the self-reported diabetes could be verified in medical files. Still, unknown diabetes could have underestimated the association between diabetes and cardiovascular mortality. It is also possible that the observed associations between diabetes treatment and cardiovascular mortality could depend on the type of diabetes. We had no information on type 1 or type 2 diabetes, but a sensitivity analysis excluding people who were diagnosed with diabetes before <40 years of age and who reported to use insulin injection suggest no large bias of the results. It should also be noted that diabetes treatment and duration is a rough estimate of diabetes severity. Unfortunately, we did not have information on glycaemic control. Nevertheless, the evidence of a dose-dependent association across diabetes treatment indicates that our classification is able to capture some of the variation in severity among people with diabetes. The physical activity questionnaire used in this study has been validated against more objective measures of fitness and activity, such as VO_2_max and ActiReg, in a subsample of young men [24]. The questionnaire was reported to have good repeatability and provide a useful measure of leisure time physical exercise. It is possible that the reported exercise volume may be overestimated due to subjective interpretations. However, if the reported exercise volume is overestimated, the presented associations between exercise and mortality is likely to be underestimated. Also, the effects of exercise may be different in young and elderly people. In the present study, only information on frequency and duration was used to classify participants. We acknowledge that exercise intensity could be of importance for cardiovascular health, but analyses stratified according to exercise intensity were not possible due to limited statistical power. Leisure time physical exercise was only measured at baseline, and information on prior changes due to diabetes diagnosis or subsequent changes throughout the follow-up period was not available. Such changes in physical activity could both attenuate and strengthen the estimated association. Also, new cases of diabetes and more people with diabetes receiving medical treatment could result in an underestimated association with mortality. Finally, a substantial proportion of participants were excluded due to missing information on physical exercise and possible selection bias cannot be ruled out.

## Conclusions

In conclusion, the results from this prospective cohort study show a positive association between diabetes severity, measured by treatment status, and mortality from cardiovascular disease and ischemic heart disease. Leisure time physical exercise was associated with reduced mortality, and this association was especially strong among people with diabetes who reported medical treatment compared to those who did not. This suggests that physical exercise should be encouraged as a therapeutic measure additional to medical treatment.

## Competing interests

No potential conflicts of interest relevant to this article were reported.

## Authors’ contributions

BM prepared and analyzed the data, interpreted the results, drafted the manuscript, and contributed to the final version of the manuscript. LBA interpreted the results and contributed to the final version of the manuscript. TILN initiated the study, interpreted the results, and contributed to the final version of the manuscript. TILN is guarantor of this work, and, as such, had full access to all the data in the study and takes responsibility for the integrity of the data and the accuracy of the data analysis. All authors read and approved the final manuscript.
